# Multimorbidity, social determinants and intersectionality in chronic patients. Results from the EpiChron Cohort

**DOI:** 10.7189/13.04014

**Published:** 2023-02-03

**Authors:** Aida Moreno-Juste, Antonio Gimeno-Miguel, Beatriz Poblador-Plou, Amaia Calderón-Larrañaga, Mabel Cano del Pozo, Maria João Forjaz, Alexandra Prados-Torres, Luis A Gimeno-Feliú

**Affiliations:** 1 EpiChron Research Group, Aragon Health Sciences Institute (IACS), IIS Aragón, Miguel Servet University Hospital, Zaragoza, Spain; 2 San Pablo Primary Care Health Centre, Aragon Health Service (SALUD), Zaragoza, Spain; 3 Network for Research on Chronicity, Primary Care, and Health Promotion (RICAPPS), Research Network on Health Services in Chronic Diseases (REDISSEC), Institute of Health Carlos III (ISCIII), Madrid, Spain; 4 Aging Research Center, Department of Neurobiology, Care Sciences and Society, Karolinska Institutet and Stockholm University, Stockholm, Sweden; 5 General Directorate of Health Care, Department of Health, Government of Aragon, Zaragoza, Spain; 6 National Center of Epidemiology, Institute of Health Carlos III (ISCIII), Madrid, Spain; 7 University of Zaragoza, Zaragoza, Spain

## Abstract

**Background:**

Multimorbidity is influenced in an interconnected way, both in extent and nature, by the social determinants of health. We aimed at implementing an intersectional approach to analyse the association of multimorbidity with five important axes of social inequality (i.e. gender, age, ethnicity, residence area and socioeconomic class).

**Methods:**

We conducted a cross-sectional observational study of all individuals who presented with at least one chronic disease in 2019 (n = 1 086 948) from the EpiChron Cohort (Aragon, Spain). Applying intersectional analysis, the age-adjusted likelihood of multimorbidity was investigated across 36 intersectional strata defined by gender, ethnicity, residence area and socioeconomic class. We calculated odds ratios (OR) 95% confidence interval (CI) using high-income urban non-migrant men as the reference category. The area under the receiver operator characteristics curve (AUC) was calculated to evaluate the discriminatory accuracy of multimorbidity.

**Results:**

The prevalence of multimorbidity increased with age, female gender and low income. Young and middle-aged low-income individuals showed rates of multimorbidity equivalent to those of high-income people aged about 20 years older. The intersectional analysis showed that low-income migrant women living in urban areas for >15 years were particularly disadvantaged in terms of multimorbidity risk OR = 3.16 (95% CI = 2.79-3.57). Being a migrant was a protective factor for multimorbidity, and newly arrived migrants had lower multimorbidity rates than those with >15 years of stay in Aragon, and even non-migrants. Living in rural vs. urban areas was slightly protective against multimorbidity. All models had a large discriminatory accuracy (AUC = 0.7884-0.7895); the largest AUC was obtained for the model including all intersectional strata.

**Conclusions:**

Our intersectional approach uncovered the large differences in the prevalence of multimorbidity that arise due to the synergies between the different socioeconomic and demographic exposures, beyond their expected additive effects.

Multimorbidity (i.e. the co-existence of two or more chronic conditions) represents a growing global health challenge that has been estimated to affect approximately 50 million people in Europe [1,2]. It is well known that multimorbidity is associated with high mortality, reduced functional status and increased use of both inpatient and ambulatory health care [3], and that it becomes progressively more common with age [1-3].

Multimorbidity is greatly influenced, both in extent and in nature, by the social determinants of health [2-6], which are defined by the World Health Organization as the social circumstances in which each person is born, grows, lives, works and gets older [7]. These determinants include, for example, socioeconomic, environmental, community as well as the health system-related factors, which are the main cause of social inequalities in the population. These differences lead to exposures and vulnerabilities that affect all aspects of individuals’ lives, including their individual health and the population [7]. The most important social factors determining health are gender, race, income, accumulated wealth, education, occupational characteristics, and social inequality based on race and ethnic group membership [8]. As a matter of fact, young and middle-aged adults living in the most deprived areas show multimorbidity prevalence rates similar to those of people aged about 10-15 years older living in the most affluent areas [3]. In addition, higher rates of multimorbidity have been found in groups with fewer educational qualifications [6], ethnic minorities [4,9,10], and in people with a low household income [4,6].

Most research and policy looking at social inequalities has analysed it as separate and disentangled phenomena [11,12]. However, more recent perspectives acknowledge the interwoven nature of social inequalities, and the non-uniform effects that the influencing context may have on populations’ health [5,11]. The intersectionality theory focuses on the understanding of social factors like gender, class and race/ethnicity/racialisation as being interconnected rather than separate, and as creating overlapping and interacting systems of discrimination or disadvantage [12-16]. Thus, the social factors conditioning the distribution of resources and power, and thus health, should be considered as interlinked rather than as unidimensional [12]. An intersectional perspective motivates the study of strata, defined by the combination of several socioeconomic dimensions, contrasting with conventional analysis of socioeconomic gradients in health often based on singular dimensions. This approach represents a new way of understanding the complex nature of health inequities [13,14], and it can improve the mapping of inequalities in health and therefore better illustrate patterns of disadvantage [12]. In addition, an intersectional approach helps us to shift the focus from individual risk factors to social power dynamics, reinforcing the importance of structural interventions that address social causes [7]. It also promotes proportionate universalism approaches that avoid “victim-blaming” as it is often done in individual approaches [11].

The concept of intersectionality in health is commonly applied to the multiple overlapping social determinants that impact the health of deprived and excluded populations, and its principles may be readily applicable in multimorbidity research. A better understanding of the epidemiology of multimorbidity is necessary to develop interventions to prevent its incidence, reduce its burden, and align health care services more closely to patients' needs [3], which may contribute to more effective public health interventions [13,16]. In this study, we aimed at implementing an intersectional approach to analyse the association between multimorbidity and five of the most relevant axes of social inequality (i.e. gender, age, ethnicity, residence area and socioeconomic class).

## METHODS

### Study design and population

We conducted a retrospective, observational study based on the EpiChron Cohort [17]. This cohort was created in 2011 for the study of chronic diseases and multimorbidity and links, at the patient level and in a pseudonymised way, the demographic and clinical information of all public health care system users in the Spanish region of Aragon. This information is collected from patients’ electronic health records (EHRs) from primary and hospital health care, pharmacy billing records, and users’ database. This open cohort is updated regularly and included at baseline the information of 1 253 292 individuals of all ages (mean age 44.2 years, 50.5% women, 11.9% migrants, 37.5% multimorbid, mean burden of 1.7 chronic diseases and 4.3 drugs). An exhaustive description of the cohort profile regarding baseline information, data sources used, and details on data curation and linkage procedures has been published elsewhere [17].

We included in the study all chronic individuals of the cohort, i.e. those who had at least one chronic disease between January 1 and December 31, 2019 (n = 1 086 948). They represented about 80% of the total population in the region (approximately 1.3 million inhabitants). To ensure the accuracy and exhaustivity of the clinical information, individuals who were not enrolled as health care system users for at least one year before January 1, 2019 were excluded. The EpiChron Cohort Study has been favourably evaluated by the Clinical Research Ethics Committee of Aragon (CEICA; protocol number PI17/0024).

### Study variables and data sources

For each subject, we analysed the following socio-demographic variables: gender, age (categorised as ≤14, 15-44, 45-64, 65-79, ≥80 years), ethnicity (non-migrant or migrant, i.e. country of birth different to Spain), length of residence in Aragon (≤15 years or >15 years), residence area (urban, i.e. people living in municipalities that concentrate at least 80% of the population of the area, and rural, i.e. the rest), and socioeconomic class using a proxy of annual gross income based on the prescription co-payment rate (low income <€18 000, medium income €18 000-€100 000, and high income >€100 000 [18,19]. Members with a mutual insurance (1.06% of patients in Aragon) were not included because information on their socioeconomic class was not available. As a way of operationalising intersectionality, we created 36 intersectional strata by combining the possible categories of each variable (i.e. 2 genders x 2 ethnicities (x 2 lengths of stay in migrants) x 2 residence areas x 3 socioeconomic classes). We used non-migrant men living in an urban area and with a high income as the reference group for the comparisons, as this group was assumed to enjoy the position of greatest structural privilege or power [12,20]. Regarding the effect of migrant status on multimorbidity, contradictory results have been found, which may depend on the length of stay and country of origin [21]; this is the reason why we also included this variable in the analyses.

Multimorbidity was operationalised as the simultaneous presence of two or more chronic conditions in a patient [1,2]. We analysed all chronic diseases registered in patients’ EHRs from primary and hospital care during the study period. Diagnoses of chronic diseases were initially coded using the International Classification of Primary Care, First Edition (ICPC-1). These codes were mapped to the International Classification System, Ninth Edition, Clinical Modification (ICD-9-CM) using a validated mapping system [22]. Diagnoses were subsequently sorted into 226 clinically relevant categories using the Clinical Classifications Software (CCS) for ICD-9-CM [23]. Conditions were classified as chronic or not using the Chronic Condition Indicator software tool [24], with a total of 153 conditions categorised as chronic. Some of the diagnostic labels were renamed by the research team to facilitate their clinical interpretation. Chronic conditions were defined as those present during the last 12 months at least, and meeting one or both of the following criteria: (i) entails limitations on self-care, independent living, and social interactions; (ii) requires ongoing interventions using medical products, services, and special equipment [25].

### Statistical analysis

Sociodemographic and clinical characteristics of the population were described using means and standard deviations (SD) for continuous variables, and frequencies and percentages for categorical variables. Differences between men and women were assessed using Student’s *t* test or χ^2^ test, as appropriate. Statistical significance was set at *P* < 0.05.

Then, an intersectional analysis to study the effect of the socioeconomic variables on the presence of multimorbidity was conducted based on the inter-categorical approach followed by Kapilashrami et al. [9] and Veenstra et al. [10]. This methodology implies moving beyond class and socioeconomic position in analysing the structural determinants of health, and on studying the strata defined by the combination of several socioeconomic dimensions. In doing so, 36 socioeconomic strata were created to analyse the interactions among the different axes of oppression-privilege, combining the two sex categories, the two geographical areas, the three income levels, and the three migrant status categories.

In this study, five consecutive logistic regression models were constructed where presence of multimorbidity was modelled as the dependent variable. We obtained odds ratios (OR) and their 95% confidence intervals (CI). The first regression model (Model 1) included only gender. The following models successively added the rest of exposures: annual gross income (Model 2), ethnicity-length of stay (Model 3), and residence area (Model 4). Finally, Model 5 included the same four variables as Model 4 and their interactions in the form of intersectional strata. All models were adjusted by age. For each model, we quantified the area under the receiver operator characteristics curve (AUC) to evaluate the discriminatory accuracy of multimorbidity. The AUC measures the accuracy of the information provided by the variables in the model to discriminate the presence/absence of multimorbidity. The AUC takes values between 0.5 and 1, where 1 indicates perfect discrimination and 0.5 means that the studied variables are not associated with multimorbidity at all [12]. We categorised the discriminatory accuracy of the model as absent or very small (AU-ROC = 0.5-0.6), small (AU-ROC = 0.6-0.7), large (AU-ROC = 0.7-0.8) or very large (AU-ROC>0.8) [12].

All the analyses were conducted in STATA software (Version 12.0, StataCorp LLC, College Station, TX, USA), with the statistical significance set at *P* < 0.05.

## RESULTS

The demographic and clinical characterisation of the 1 038 307 patients included in the study (53.7% women, mean age 53.9 years) is shown in **Table 1**. The majority were non-migrants (87.4%), lived in urban areas (60.1%), and had a low annual income (71.4%). A higher proportion of women belonged to the low-income group compared to men.

**Table 1:** Characteristics of the study population

	Men	Women	Total	*P*-value‡
**Population**, n (%)	481 008 (46.33)	557 299 (53.67)	1 038 307 (100.00)	
**Age** **in years**, mean (SD)	52.88 (23.70)	54.77 (24.09)	53.90 (23.93)	<0.001
**Age group in years**, n (%)				<0.001
0-14	38 918 (8.09)	34 115 (6.12)	73 033 (7.03)	
15-44	127 751 (26.56)	157 115 (28.19)	284 866 (27.44)	
45-64	152 822 (31.77)	164 007 (29.43)	316 829 (30.51)	
65-79	91 570 (19.04)	99 275 (17.81)	190 845 (18.38)	
≥80	69 947 (14.54)	102 787 (18.44)	172 734 (16.64)	
**Ethnicity**, n (%)				<0.001
Non-migrant	424 153 (88.18)	483 289 (86.72)	907 442 (87.40)	
Migrant	56 855 (11.82)	74 010 (13.28)	130 865 (12.60)	
**Length of stay in Aragon**, n (%)				<0.001
≤15years	42 309 (74.42)	58 041 (78.42)	100 350 (76.68)	
>15 years	14 546 (25.58)	15 969 (21.58)	30 515 (23.32)	
Residence area*, n (%)				<0.001
Urban	281 728 (58.57)	342 690 (61.49)	624 418 (60.14)	
Rural	199 278 (41.43)	214 604 (38.51)	413 882 (39.86)	
**Annual gross income†**				<0.001
Low	312 734 (65.02)	428 813 (76.94)	741 547 (71.42)	
Medium	165 633 (34.43)	126 662 (22.73)	292 295 (28.15)	
High	2641 (0.55)	1824 (0.33)	4465 (0.43)	
**Number of chronic diseases**, mean (SD)	3.41 (2.50)	4.00 (2.77)	3.73 (2.67)	<0.001
**Multimorbidity**, n (%)	354 552 (73.71)	448 865 (80.54)	803 417 (77.38)	<0.001
**Number of chronic diseases**, n (%)				<0.001
1	126 456 (26.29)	108 434 (19.46)	234 890 (22.62)	
2	99 335 (20.70)	100 625 (18.10)	199 960 (19.30)	
3	72 383 (15.05)	83 023 (14.90)	155 406 (14.97)	
4	54 133 (11.25)	67 005 (12.02)	121 138 (11.67)	
5	40 616 (8.44)	54 784 (9.83)	95 400 (9.19)	
6	29 802 (6.20)	43 384 (7.78)	73 186 (7.05)	
7	20 857 (4.34)	33 082 (5.94)	53 939 (5.19)	
>8	37 426 (7.78)	66 962 (12.02)	104 388 (10.05)	

SD – standard deviation

*Residence area: urban, i.e. people living in municipalities that concentrate at least 80% of the population of the area, and rural, i.e. the rest.

†Annual gross income based on the prescription co-payment rate (low income <€18 000, medium income €18 000-€100 000, and high income >€100 000).

‡*P*-values for the comparisons between men and women (using Student’s *t* test or χ^2^ test, as appropriate).

**Figure 1:**
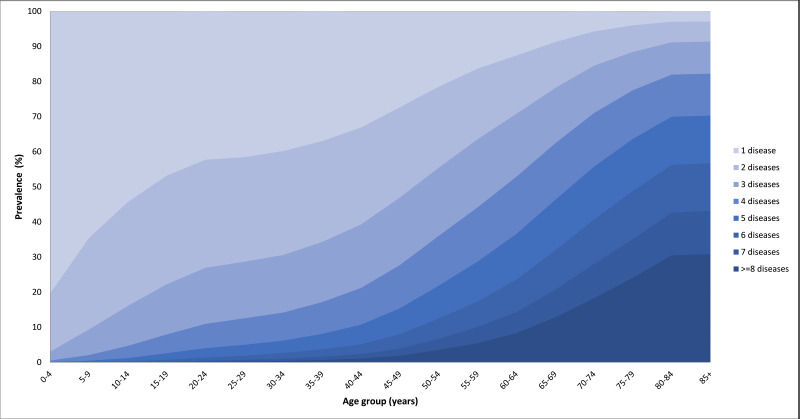
Number of chronic diseases by age group.

**Figure 2:**
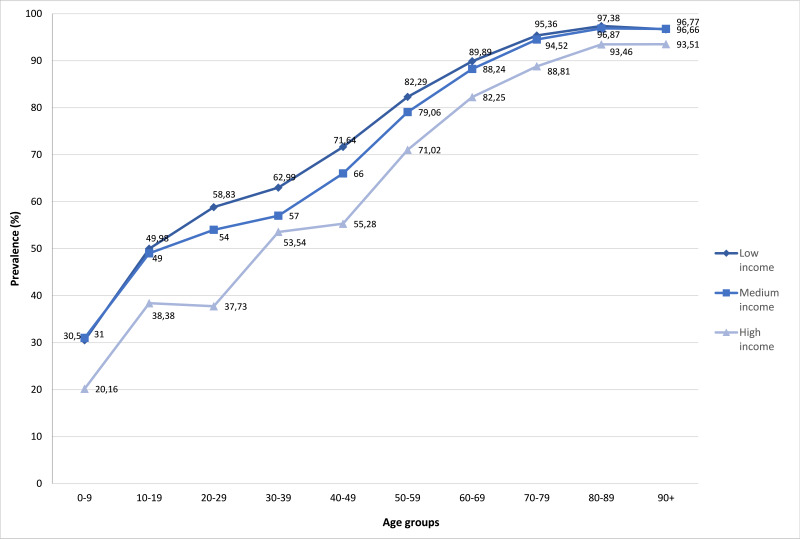
Prevalence of multimorbidity by age group and annual gross income. Annual gross income based on the prescription co-payment rate (low income <€18 000, medium income €18 000-€100 000, and high income >€100 000).

Around three in four individuals with at least one chronic disease had multimorbidity, with a mean disease burden of nearly four comorbidities. Both the mean number of diseases and the prevalence of multimorbidity substantially increased with age (**Figure 1** and **Figure 2**). Individuals over 90 years of age showed a prevalence of multimorbidity of 95%, and 30% of them had more than eight chronic diseases registered in their EHR.

A clear gradient between income level and prevalence of multimorbidity was found. As shown in **Figure 2**, the high-income group presented with lower prevalence rates of multimorbidity than the rest at all ages, although differences tended to disappear at older ages. The low-income group presented with slightly higher prevalence rates of multimorbidity than the middle-income one, especially in the middle ages of life. Young and middle-aged low-income individuals showed rates of multimorbidity equivalent to those of high-income people aged about 20 years older.

As shown in Models 1, 2, 3 and 4, being a woman and belonging to the low-income group increased the likelihood of multimorbidity (**Table 2**). By contrast, being migrant (especially with ≤15 years of residence), belonging to the high-income group and living in rural areas was associated with a lower likelihood of multimorbidity.

**Table 2:** Likelihood of multimorbidity based on sociodemographic factors

	Model 1*	Model 2	Model 3	Model 4
	OR (95% CI)	OR (95% CI)	OR (95% CI)	OR (95% CI)
**Gender**				
Men	Reference	Reference	Reference	Reference
Women	1.49 (1.48-1.51)	1.47 (1.46-1.49)	1.48 (1.46-1.49)	1.47 (1.46-1.49)
**Annual gross income**				
Low		1.14 (1.13-1.16)	1.19 (1.18-1.21)	1.20 (1.18-1.21)
Middle		Reference	Reference	Reference
High		0.65 (0.60-0.70)	0.65 (0.60-0.70)	0.65 (0.60-0.70)
**Ethnicity-residence length**				
Native			Reference	Reference
Immigrant ≤15 years			0.74 (0.73-0.75)	0.74 (0.73-0.75)
Immigrant >15 years			0.96 (0.93-0.99)	0.96 (0.93-0.99)
**Residence area**				
Urban				Reference
Rural				0.96 (0.95-0.97)
**AUC**	0.7878	0.7884	0.7887	0.7888

OR – odds ratio, CI – confidence interval, AUC – area under the receiver operating characteristics curve

*ORs with 95% CIs and AUC values obtained from four consecutive logistic regressions modelling the presence of multimorbidity as a function of four variables. All models were adjusted for age.

**Figure 3:**
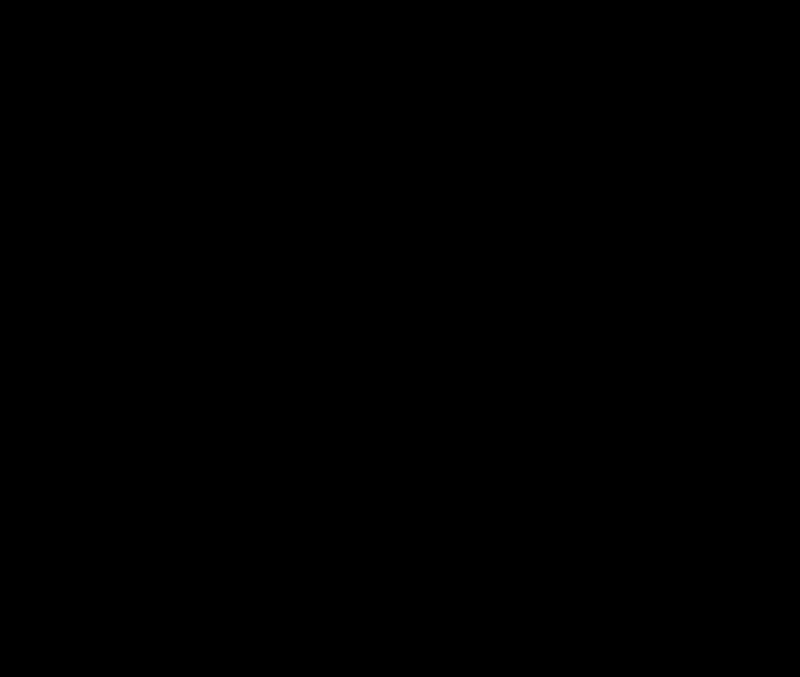
Likelihood of multimorbidity as a function of intersectional strata derived from all sociodemographic factors using one single logistic regression model (Model 5), which analysed gender, annual gross income, ethnicity-length of stay, residence area, and their interactions in the form of intersectional strata. Odds ratios (OR) and 95% confidence intervals (CI) were calculated. All models were adjusted for age. Model area under the receiver operating characteristics curve (AUC) = 0.7895. In case of women/immigrant >15 years /rural/high-income, the OR could not be obtained in this stratum due to the small number of observations.

Model 5 showed a clear intersectional gradient in the relationship among the studied variables (**Figure 3**). The highest likelihood of multimorbidity occurred in the group of low-income migrant women living in urban areas of Aragon for >15 years, in whom the odds was 3.2 times higher compared with high-income non-migrant urban men (reference group). The analysis of intersectional strata revealed a lower likelihood of multimorbidity in men compared with women, regardless of migration status, annual gross income, and residence area. In fact, all low- and middle-income women (regardless of ethnicity and residence area, with the exception of high-income migrant women living in rural areas of Aragon for ≤15 years) presented with twice the odds of multimorbidity than the reference group.

All models had a large discriminatory accuracy, which increased slightly across the successive models and was largest when all intersectional strata were simultaneously included in the model (i.e. Model 5).

## DISCUSSION

The prevalence of multimorbidity in our study population was strongly related to the social determinants of health. An intersectional view helped us uncover the large differences that arise from the interconnections between the different axes of social inequality, and it revealed that the impact of the before-mentioned factors on the outcome were affected by each other; in other words, we found evidence of potential synergies among exposures [12]. Low-income immigrant women living in urban areas of Aragon for >15 years were particularly disadvantaged. In contrast, the most advantaged group was that of high-income native males residing in urban areas. As in Wemrell's study [12], being female, poor and a long-stay migrant epitomises the impact of the social determinants of health on people's lives.

The characterisation of our study population was similar to other studies of people with chronic health conditions [3]. We observed that men and women were equally represented. Multimorbidity increased with age; the strong association of multimorbidity with age is well recognised [3], with higher multimorbidity prevalence in older people than in young or middle-aged people [3,26]. But, as it is known [3,27], multimorbidity did not only affect older adults; it was also a common finding in young people.

In the univariate analyses, being a woman was also found to be a clear known risk factor, and in the intersectional analysis, women presented with multimorbidity more frequently than men across all strata. In developed countries, women have generally worse health than men [5,13], particularly in terms of mental health, related to biological, behavioural and psychological differences between men and women, and patriarchy that reduces women's access to material resources [14]. Other studies also observed that all strata comprising women had a higher average risk of worse health than those including males with the same income and immigration status [12]. Differences in income have been shown to have a greater impact on the health of women compared with men [9]. This is in correspondence with the foundational insight of intersectionality scholarship, which states that gendered issues are fundamentally mediated by factors including racialisation and class [12]. Moreover, individual income produces important gender inequalities in health against women, caused by unstable employment or low payment [5], less secure and more informal work with precarious employment conditions, and minimal regulation and social protection [9].

Social class inequalities have been widely studied in the literature, showing that individuals in lower socioeconomic positions have higher risk of morbidity and earlier mortality than those in higher socioeconomic positions [5], as we also saw in our study. In addition, we found that the income gradient decreasingly influenced the risk of multimorbidity as age increased. Young and middle-aged adults with low- and middle-income started with high rates of multimorbidity almost 20 years earlier than patients with high income. Socioeconomic variables should be understood as the primary drivers of health inequalities, drawing on the concept of intersectionality to argue for a more complex understanding of identity, social position and inequality in the social determinants of health [9]. This would confirm that the negative biological and social consequences of low income can accumulate across the life course and contribute to an increased risk of developing chronic diseases. Wermell et al. evaluated the socioeconomic differences in excess risk of type 2 diabetes and explained these differences by established risk factors such as overweight, physical inactivity, smoking, and heredity in combination with psychosocial factors [13]. A metanalysis observed an inconsistent association between income and multimorbidity, which might reflect differences in populations and settings, but they found that this association varied by age and gender [6]. Barnett et al. also noted an excess of multimorbidity in young and middle-aged adults living in the most deprived areas of Scotland, with prevalence rates that were comparable to those of people aged about 10-15 years older living in the most affluent areas [3].

Differences in the health status of different ethnic groups are often comparable in magnitude to socioeconomic health inequalities [9]. In our study, being a migrant was a protective factor of multimorbidity, and newly arrived migrants had a lower risk of multimorbidity than those with more than 15 years of stay in Aragon and non-migrants. This was confirmed in the intersectional analysis, and it could be a consequence of the so-called “healthy migration effect”. Numerous studies showed how the migrant population has a high level of health on arrival in the host countries, even higher than the host population. However, this effect fades away over the years of stay, probably due to the adverse living conditions in which these people live [21,28-32]. In our cohort, most of the migrants had lived in Aragon for less than 15 years; thus, migrants with high health levels were predominant. Studies including immigrants with a longer length of residence were more likely to detect the dynamism of the impact of migrant status over time [21,28,30-33]. In other studies, immigrants had a higher risk of poor self-reported health compared to natives with the same income and gender, but these studies failed to account for length of residence [12]. Immigrants’ health disadvantage is often explained by their lower socioeconomic status or their experience of discrimination [9,13,14], involuntary displacement, and living in socioeconomically vulnerable areas [13], especially if a low socio-economic level and minority ethnic status coexist [9].

Individuals’ health status can also differ depending on geographical contexts [5]. In our study, residing in a rural area was slightly protective against multimorbidity in comparison to living in urban areas, among patients with equal gender, ethnicity and annual gross income, with the exception of middle-income native rural women, high-income native rural men, and middle-income immigrant rural men living >15 years in Aragon. Indeed, psychosocial factors play an important role when explaining gender inequalities across social class and regional development [5]. Bisquera et al. stated in 2021 that urban environments were generally characterised by deprivation across multiple domains, a high proportion of ethnic minority and migrant populations, and by reduced life expectancy and life satisfaction [1]. However, a metanalysis in 2021 reported mixed results regarding the relationship between multimorbidity and rurality [4].

The high discriminatory accuracy of the models combining information on gender, annual gross income, ethnicity and residence area provides proof-of-concept to the use of intersectional analysis in the combined assessment of the influence of social inequality axes on multimorbidity. This intersectional vision is useful for health care professionals. It reminds us that each patient's life is crossed by the dynamics of the different axes of power-inequality and that we must assess their situation with a holistic biopsychosocial approach [34]. The information gathered in our study could be relevant for planning more effective public health interventions, following the idea that public health and preventive medicine actions must be universal, not targeted, but with a scale and intensity that is proportional to the level of disadvantage of the recipients (i.e. proportionate universalism) [13]. An intersectional approach in public health is also critical for research and teaching to highlight the health disparity effects and the underlying structures that create and maintain them [15]. This intersectional approach also reminds us of the importance of proposing social solutions to health problems and their social causes [7,13,14].

### Strengths and limitations

The main strength of this study is the inclusion of all chronic patients (approximately 80% of the total population in Aragon) from the reference population. Moreover, we exhaustively studied multimorbidity by including a total of 114 chronic diagnoses that were extracted from patients’ EHRs of both primary and hospital care, and not only the most prevalent or severe ones as is common practice in the field. The fact that there is no standard approach for the selection and definition of morbidities leads inevitably to heterogenous operationalisations of multimorbidity that are highly dependent on data availability [3]. Last, data from the EpiChron Cohort undergoes continuous quality controls that ensure its accuracy and reliability for research purposes [17].

The main limitation inherent to cross-sectional, observational studies is the inability to establish causal relationships between exposures and outcome [12]. Multimorbidity was defined as the presence of two or more chronic diseases, which does not take account of the varying presentations and effects that different combinations of conditions and their severity may have on individuals [26]. We also lacked information on some relevant variables like the educational level. In addition, we only had available information of patients with at least one chronic disease, which prevents us from providing population-based prevalence estimates. Future studies should follow a population-based approach and use alternative multimorbidity operationalisations, which may enlarge the differences observed across intersectional strata. Intersectionality theory originates from qualitatively and theoretically oriented research, and some researchers have questioned its commensurability using quantitative analysis [35]. On the other hand, other authors have highlighted the importance of applying intersectional approaches in quantitative population health research [12].

## CONCLUSIONS

Multimorbidity is considered a public health priority and, therefore, its characterisation is relevant for health systems. We found that multimorbidity increased with age, female gender and lower income. Young and middle-aged low-income individuals showed rates of multimorbidity equivalent to those of high-income people aged about 20 years older. In addition, our intersectional approach uncovered the large differences in the prevalence of multimorbidity that arise due to the synergies between the different socioeconomic and demographic exposures, beyond their expected additive effects.
